# Local estrogen axis in the human bone microenvironment regulates estrogen receptor-positive breast cancer cells

**DOI:** 10.1186/s13058-017-0910-x

**Published:** 2017-11-15

**Authors:** Derek F. Amanatullah, John S. Tamaresis, Pauline Chu, Michael H. Bachmann, Nhat M. Hoang, Deborah Collyar, Aaron T. Mayer, Robert B. West, William J. Maloney, Christopher H. Contag, Bonnie L. King

**Affiliations:** 1 Department of Orthopaedic Surgery, Stanford University School of Medicine, 450 Broadway Street, Pavilion C, 4th Floor, Redwood City, CA 94063-6342 USA; 2 Department of Biomedical Data Science, Stanford University School of Medicine, Redwood Building, Room T101F (MC 5405), Stanford, CA 94305 USA; 3 Department of Pathology, Stanford University School of Medicine, Edwards, Room 264, 1291 Welch Road, Stanford, CA 94305-5324 USA; 4 Department of Pediatrics, Stanford University School of Medicine, 150E Clark Center, 318 Campus Drive, Stanford, CA 94305-5427 USA; 5 Research IT, Stanford University School of Medicine, 3172 Porter Drive, Palo Alto, CA 94304 USA; 6 Patient Advocates in Research (PAIR), Danville, CA 94506 USA; 7 Department of Bioengineering, Stanford University School of Medicine, 153E Clark Center, 318 Campus Drive, Stanford, CA 94305 USA; 8 Present address: Departments of Biomedical Engineering, and Microbiology & Molecular Genetics, Institute for Quantitative Health Science and Engineering, Michigan State University, 775 Woodlot Dr, East Lansing, MI 44823 USA

**Keywords:** Estrogen receptor positive breast cancer, Breast cancer metastasis to bone, Aromatase, Aromatase inhibitors, Bone tissue culture

## Abstract

**Background:**

Approximately 70% of all breast cancers express the estrogen receptor, and are regulated by estrogen. While the ovaries are the primary source of estrogen in premenopausal women, most breast cancer is diagnosed following menopause, when systemic levels of this hormone decline. Estrogen production from androgen precursors is catalyzed by the aromatase enzyme. Although aromatase expression and local estrogen production in breast adipose tissue have been implicated in the development of primary breast cancer, the source of estrogen involved in the regulation of estrogen receptor-positive (ER+) metastatic breast cancer progression is less clear.

**Methods:**

Bone is the most common distant site of breast cancer metastasis, particularly for ER+ breast cancers. We employed a co-culture model using trabecular  bone tissues obtained from total hip replacement (THR) surgery specimens to study ER+ and estrogen receptor-negative (ER-) breast cancer cells within the human bone microenvironment. Luciferase-expressing ER+ (MCF-7, T-47D, ZR-75) and ER- (SK-BR-3, MDA-MB-231, MCF-10A) breast cancer cells were cultured directly on bone tissue fragments or in bone tissue-conditioned media, and monitored over time with bioluminescence imaging (BLI). Bone tissue-conditioned media were generated in the presence vs. absence of aromatase inhibitors, and testosterone. Bone tissue fragments were analyzed for aromatase expression by immunohistochemistry.

**Results:**

ER+ breast cancer cells were preferentially sustained in co-cultures with bone tissues and bone tissue-conditioned media relative to ER- cells. Bone fragments analyzed by immunohistochemistry revealed expression of the aromatase enzyme. Bone tissue-conditioned media generated in the presence of testosterone had increased estrogen levels and heightened capacity to stimulate ER+ breast cancer cell proliferation. Pretreatment of cultured bone tissues with aromatase inhibitors, which inhibited estrogen production, reduced the capacity of conditioned media to stimulate ER+ cell proliferation.

**Conclusions:**

These results suggest that a local estrogen signaling axis regulates ER+ breast cancer cell viability and proliferation within the bone metastatic niche, and that aromatase inhibitors modulate this axis. Although endocrine therapies are highly effective in the treatment of ER+ breast cancer, resistance to these treatments reduces their efficacy. Characterization of estrogen signaling networks within the bone microenvironment will identify new strategies for combating metastatic progression and endocrine resistance.

**Electronic supplementary material:**

The online version of this article (doi:10.1186/s13058-017-0910-x) contains supplementary material, which is available to authorized users.

## Background

Estrogen receptor-alpha (ER-α), which mediates the action of estradiol in fueling the development and progression of breast cancer, is expressed in approximately 70% of all malignant breast tumors, making estrogen receptor-positive (ER+) breast cancer the most common breast cancer subtype [1]. ER-α functions as an estrogen ligand-activated transcription factor to regulate a variety of genes that govern cell proliferation and survival in normal tissues and tumors [2, 3]. Estrogen production is catalyzed by the P450 cytochrome aromatase enzyme, which converts the androgens testosterone and androstenedione into estradiol and estrone, respectively [4, 5]. While the ovaries are the primary source of estrogens in premenopausal women, most breast cancers are diagnosed during the postmenopausal years, when the ovaries no longer provide significant systemic levels of this hormone [6]. In postmenopausal women, the largest source of estrogen is adipose tissue [4], and local production by breast adipose tissue has been linked to the development of primary breast cancer [7–11]. In addition, the link between breast cancer and obesity has led to the hypothesis that peripheral production of estrogen by subcutaneous and abdominal fat depots drives the development and progression of breast cancer [12–15]. However, estrogens are also produced in a variety of other tissues, including the brain, vasculature smooth muscle, and bone, where they act locally in paracrine fashion [5].

ER+ breast cancers are effectively treated with three types of endocrine therapies: (i) selective estrogen receptor modulators (SERMs), which block estrogen access to the estrogen receptor, (ii) selective estrogen receptor downregulators (SERDS), which downregulate the estrogen receptor, or (iii) aromatase inhibitors (AIs), which inhibit the production of estrogen [16–18]. However, a significant number of women have intrinsic and acquired resistance to these therapies. Among patients with early-stage breast cancer who receive adjuvant endocrine therapies, 25% of cancers will recur within 10 years [16–18]. In the metastatic setting, endocrine therapies mitigate tumor progression in less than 50% of patients, with resistance and progression ultimately occurring in all patients [16–18]. Thus, circumventing endocrine resistance is critical to achieving lasting responses to endocrine therapy. Many aspects of the estrogen axis, including estrogen biosynthesis, target cell responses, and modulation by endocrine therapies exhibit tissue-specific patterns. A notable example is the pleiotropic nature of the SERM tamoxifen, which acts as an antagonist in the breast, but as an agonist in the uterus. These tissue-specific patterns are presumably generated by microenvironmental factors that interact with the estrogen signaling network [19]. Because metastasis causes most breast cancer deaths, understanding breast cancer progression and response to endocrine therapies within the context of the metastatic microenvironment is vitally important.

Bone is the most common site of breast cancer metastasis, particularly in ER+ patients [20–23]. We previously developed a co-culture model platform using human bone tissues obtained from total hip replacement (THR) surgeries to study dynamic breast cancer cell behaviors, including migration, colonization, and proliferation, within the native 3-dimensional bone tissue microenvironment [24–26]. Here we present data showing that these tissues preferentially sustain ER+ breast cancer cells relative to estrogen receptor-negative (ER-) breast cancer cells in short-term co-culture, and that pretreatment of bone tissues with aromatase inhibitors reduces local estrogen levels and ER+ breast cancer cell proliferation during co-culture. These findings suggest that ER+ breast cancer cells are regulated by estrogen and/or other factors produced locally within the bone tissue microenvironment. Characterization of this signaling axis within the bone metastatic niche may reveal new approaches for managing the metastatic progression of the most common clinical subtype of breast cancer.

## Methods

### Bone tissue collection and processing

**Fig. 1:**
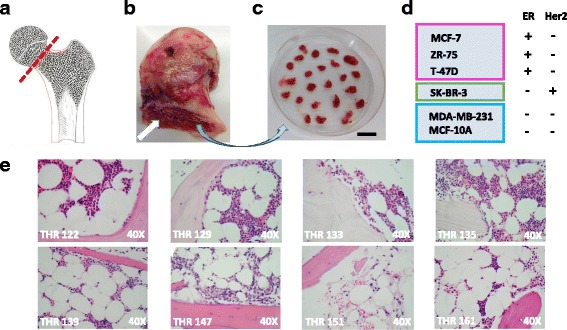
Human bone tissue co-culture model. **a** Femoral head removal during total hip replacement (THR) surgery (image credit: Moran & Rowley – Visualhistology.com). **b** Discarded femoral head exposing trabecular bone (white arrow). **c** Isolated trabecular bone tissue fragments (bar = 3 mm). **d** Panel of breast cancer cell lines transfected with luciferase and enhanced green fluorescence protein (EGFP): estrogen receptor-positive (ER+) MCF-7, ZR-75 and T-47D, ER-/human epidermal growth factor receptor 2 (Her2) + SK-BR-3, and ER- MDA-MB-231 and MCF-10A cells. **e** Hematoxylin and eosin stained histologic sections of trabecular bone tissues from a subset of THR specimens illustrating mineralized and marrow compartments

All surgical specimens and patient medical record information, including diagnoses and disease histories (obtained from the STRIDE database by Research IT, Stanford Medicine), were collected under Institutional Review Board (IRB) protocol #38625 in accordance with the regulations of the Stanford University Research Compliance Office. Femoral heads discarded after removal during elective THR were collected following surgeries performed in the Department of Orthopaedic Surgery at the Stanford University Medical Center. Specimens were collected from 28 male and female surgical patients over a period of 17 months. Femoral head specimens were placed in physiological saline, transported to the lab immediately following surgery, and placed into a Pyrex dish (VWR, Radnor, PA, USA). Using a sterile glove to hold the femoral head with one hand, trabecular bone fragments measuring ~3–5 mm^2^ were dissected from the femoral head and neck using a surgical Rongeur (Fine Science Tools, Foster City, CA, USA) for placement into culture dishes as described previously [24–26]. For a subset of specimens, representative pieces of trabecular bone were also fixed at the time of isolation in 10% formalin, decalcified, embedded in paraffin, and sectioned for hematoxylin and eosin (H&E) staining (Fig. 1e).

### Breast cancer cell lines

ER+ MCF-7, T-47D, ZR-75-1, and ER- SK-BR-3, MDA-MB-231 breast cancer and MCF-10A breast cell lines were purchased from the American Type Culture Collection (ATCC; Rockville, MD, USA) and engineered for the stable expression of firefly luciferase and enhanced green fluorescence protein (EGFP) using the Sleeping Beauty transposon on the plasmid pKT2/LuBiG that was co-transfected with the transposase plasmid pK/hUbiC-SB11 [27, 28]. The resulting transfected cell lines MCF-7-FLuc-EGFP, T-47D-FLuc-EGFP, ZR-75-1-FLuc-EGFP, SK-BR-3-FLuc-EGFP, MDA-MB-231-FLuc-EGFP, and MCF-10A-FLuc-EGFP are referred to by their parental name, without the additional designation FLuc-EGFP throughout the manuscript and figures. All cell lines were authenticated by short tandem repeat (STR) profiling following transfection (Genetica Cell Line Testing, Burlington, NC, USA), and tested negative for mycoplasma using the MycoAlert® Mycoplasma Detection Kit (Lonza, Rockland, ME, USA).

### Media and cell/tissue culture reagents

Cell lines were routinely maintained in the following types of media containing fetal bovine serum (FBS): DMEM-10% FBS (MCF-7, MDA-MB-231), RPMI-10% FBS (T-47D, ZR-75), McCoys-10% FBS (SK-BR-3) (Gibco, Life Technologies, Grand Island, NY, USA) or Clonetics MEBM + SingleQuot supplements (MCF-10A) (Lonza, Walkersville, MD, USA), plus blasticidin (InvivoGen, San Diego, CA, USA). Experiments, with one exception, were performed in DMEM-10%FBS +/− treatment agents. A subset of experiments was performed in phenol red-free medium containing 5% or 10% charcoal-stripped FBS (Gibco, Life Technologies, Grand Island, NY, USA). Letrozole (Sigma-Aldrich, St. Louis, MO, USA) was prepared as a 50-mM stock solution in 70% ethanol and stored at 4 °C. Anastrozole and exemestane (Selleck Chemicals, Houston, TX, USA) were purchased as 10-mM stock solutions in dimethylsulfoxide (DMSO), and aliquots were stored at −20 °C. Water-soluble 17β-Estradiol (Sigma-Aldrich, St. Louis, MO) was prepared as a 4.29-mM solution in water and stored as aliquots at −20 °C. Testosterone (Sigma-Aldrich, St. Louis, MO, USA) was purchased in compliance with the Stanford University controlled substance program, and prepared as a 1.7-mM stock solution in 70% ethanol stored at 4 °C.

### Breast cancer cell/bone tissue co-culture

**Fig. 2:**
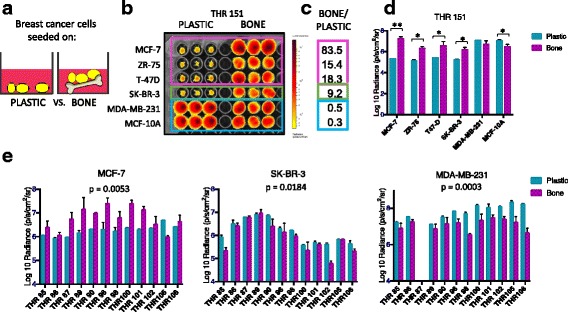
Human bone tissue fragments preferentially sustain estrogen receptor-positive (ER+) breast cancer cell viability in short-term co-culture. **a** Experimental design in which breast cancer cell suspensions were seeded into plastic wells or directly onto bone tissue fragments. **b** Bioluminescence imaging (BLI) of plate containing six breast cancer cell lines (ER+ MCF-7, ZR-75 and T-47D, ER-/human epidermal growth factor receptor 2 (Her2) + SK-BR-3, and ER- MDA-MB-231 and MCF-10A) on day 4 of culture shows signal generated during culture in plastic wells vs. bone tissue fragments isolated from total hip replacement (THR) specimen THR 151. **c** Ratio of averaged triplicate BLI signal following culture on bone vs. plastic for each cell line shown in (**b**), revealing highest ratios for the ER+ breast cancer cell lines MCF-7 (83.5x), ZR-75 (15.4x), and T-47D (18.3x), an intermediate ratio for ER-/HER2+ SK-BR-3 cells (9.2x), and lowest ratios for the ER- MDA-MB-231 (0.5x) and MCF-10A (0.3x) breast cells. **d** Averaged triplicate BLI signal detected for each cell line growing on plastic vs. bone shown in (**b**). BLI signal was significantly greater following culture on bone vs. plastic for the ER+ breast cancer cell lines MCF-7 (**p < 0.001), ZR-75 (*p < 0.01), T47-D (*p < 0.05), and the ER-/Her2-positive cell line SK-BR-3 (*p < 0.01), not different for the ER- MDA-MB-231 cells, and was significantly reduced for the ER- MCF-10A cells (*p < 0.05), as determined by *t* test (*n* = 3, error bars represent standard deviation). **e** Averaged triplicate BLI signal generated by MCF-7, SK-BR-3, and MDA-MB-231 cells cultured on plastic vs. bone fragments from a series of 12 THR surgical specimens. Signal was significantly greater following culture on bone vs. plastic for ER+ MCF-7 cells (*p* = 0.0053), whereas it was significantly reduced for ER- SK-BR-3 cells (*p* = 0.0184) and MDA-MB-231 cells (*p* = 0.0003) as determined by ANOVA (*n* = 3, error bars represent standard deviation). These results confirm that bone tissues from multiple patients preferentially sustain ER+ breast cancer cell viability in culture relative to ER- breast cancer cells

Two different co-culture formats were used. In the first format, illustrated schematically in Fig. 2a, breast cancer cell responses were compared following direct seeding onto bone tissue fragments vs. into plastic culture wells as follows. Trabecular bone tissue fragments measuring ~ 3 mm in diameter were placed into the empty wells of a 96-well tissue culture plate (one fragment/well, in triplicate for each treatment or control variable). Cell suspensions delivering 1–6 × 10^3^ breast cancer cells suspended in 10-μL volumes of DMEM-10% FBS were pipetted directly onto the top of each bone fragment, and into empty adjacent wells. Following seeding, the plate was incubated in a CO_2_ humidified 37 °C tissue culture incubator with 5% CO_2_ for 45 min to promote cell attachment. Next, 200 μL DMEM-10% FBS was added to all wells prior to continued culture in a CO_2_ humidified tissue culture incubator for 4 days, at which time relative breast cancer cell numbers were assessed by bioluminescence imaging (BLI).

**Fig. 3:**
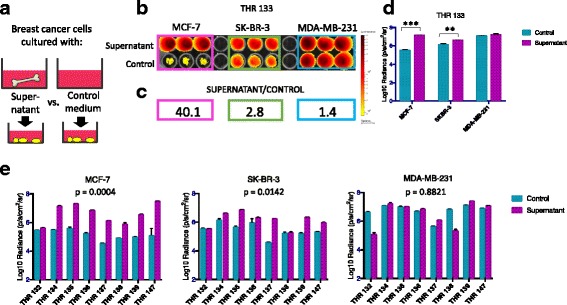
Conditioned media from cultured human bone tissue fragments preferentially stimulate estrogen receptor-positive (ER+) breast cancer cell proliferation. **a** Experimental design in which breast cancer cells were seeded into tissue culture wells containing bone tissue-conditioned supernatants vs. control medium (DMEM-10%FBS). **b** Bioluminescence imaging (BLI) on day 4 of culture showing signal for ER+ MCF-7, and ER- SK-BR-3 and MDA-MB-231 breast cancer cells cultured in supernatants generated with total hip replacement (THR) specimen 133 vs. control medium. **c** Ratio of averaged triplicate BLI signal on day 4 of culture in supernatant vs. control medium for each cell line shown in (**b**), revealing the highest ratio for ER+ MCF-7 (40.1x), and lower ratios for ER- SKBR-3 (2.8x) and MDA-MB-231 (1.4x) cells. **d** Averaged triplicate BLI signal detected for each cell line following culture in supernatant vs. control medium shown in (**b**). BLI signal was significantly greater during culture with bone tissue supernatants vs. control medium for ER-positive MCF-7 (***p = 0.0000028) and ER-/human epidermal growth factor receptor 2 (Her2)-positive SK-BR-3 (**p = 0.0046), but not ER- MDA-MB-231 cells, as determined by *t* test (*n* = 3, error bars represent standard deviation). **e** Averaged triplicate BLI signal generated by MCF-7, SK-BR-3, and MDA-MB-231 cells cultured in control vs. conditioned media generated from a series of eight THR surgical specimens. Analysis of variance demonstrated that BLI signal for cells cultured in conditioned vs. control media was dramatically increased for MCF-7 cells (*p* = 0.0004), moderately increased for SK-BR-3 cells (*p* = 0.0142), but was not changed for MDA-MB-231 cells (*p* = 0.8821). These results confirm that conditioned media generated from multiple patient specimens preferentially promote the proliferation of ER+ breast cancer cells

In the second format, illustrated schematically in Fig. 3a, bone tissue fragments were cultured to generate conditioned medium supernatants, which were subsequently used to culture breast cancer cells as follows. To address the issue of tissue fragment variability, five similarly sized trabecular bone tissue fragments/well were cultured in 6-well tissue culture plates containing 5 mL of DMEM-10% FBS +/− treatments per well for 48 h. Additional experiments were performed in phenol red-free medium with charcoal-stripped serum. Treatments included the addition of the aromatase inhibitors letrozole (0, 10 nM, 100 nM, 10 μM, 100 μM), anastrozole (50 nM) or exemestane (100 nM), and testosterone (10 nM). Following culture, supernatants were collected, filtered through a 70-μM strainer and centrifuged to pellet debris. Supernatants were subsequently used to culture breast cancer cells seeded in triplicate wells in 96-well culture plates. Aliquots of supernatants from a subset of cultures were also frozen at −80 °C for enzyme-linked immunosorbent assay (ELISA).

### ELISA

The 17β-estradiol levels in bone tissue-conditioned supernatants and control media were analyzed using two different commercially available competitive ELISAs according to the manufacturers’ recommended protocols. The first (B-Bridge Estradiol Immunoassay Kit, B-Bridge International Inc., Santa Clara, CA, USA) had a sensitivity of 39.6 pg/mL and limit of detection of 26.5 pg/mL. The second (Cayman Chemical Estradiol ELISA Kit, Cayman Chemical, Ann Arbor, MI, USA) had a sensitivity of 15 pg/mL and lower detection range of 6.6 pg/mL. Aliquots of frozen supernatants generated as described above were analyzed for each experimental and control condition, including bone tissue-conditioned media generated in the presence vs. absence of aromatase inhibitors or testosterone, and control media (DMEM-10%FBS or phenol red-free medium-10% charcoal-stripped FBS) subjected to similar conditions as the experimental cultures. Standard curves were generated using control media, and undiluted aliquots of each sample were analyzed in duplicate (B-Bridge kit) or triplicate (Cayman kit). Plates were analyzed by spectrophotometry for absorbance and read at 540 nm to generate optical density (OD) values. Standard curves and sample values were plotted and estradiol values were determined through interpolation using GraphPad Prism Software, Version 7.0c (B-Bridge kit), or using the Cayman data analysis tool.

### Bioluminescence imaging

Bioluminescence imaging was performed on cells and tissues in an IVIS 50 Imaging device (Perkin Elmer, Hopkinton, MA, USA) immediately following addition of D-luciferin substrate (150 μg/mL) to all wells. Imaging parameters were F-stop of 1, small binning, level D, and exposure time of 5 min. The signal intensities are reported, in most cases, as average log 10 radiance (photons/second/cm^2^/steradian), but in some cases as radiance (photons/second/cm^2^/steradian). Average radiance was integrated over individual wells designated as regions of interest (ROI) using the Living Image Software Program (Version 4.3.1., Perkin Elmer).

### Immunohistochemical analysis

Trabecular bone tissue fragments were fixed in 10% buffered formalin, decalcified in buffered Versanate (American Mastertech, Lodi, CA, USA) and embedded in paraffin for preparation as H&E or immunostained sections. Breast cancer cell line suspensions were fixed in 10% buffered formalin, resuspended in liquid Histogel (Richard-Allan Scientific, Kalamazoo, MI, USA) and embedded in paraffin after solidification. Five-micron-thick sections were transferred to slides and processed for immunohistochemical staining. Placental tissue (aromatase positive control) slides were purchased from Abcam (Cambridge, MA, USA). After deparafinization, endogenous peroxidase was blocked with 3% hydrogen peroxide. Antigen retrieval was performed in a microwave using Antigen Target Retrieval Solution (pH9) (Agilent Technologies, Santa Clara, CA, USA) heated to 100 °C for 10 min. Staining was performed using a 1:100 dilution of the mouse monoclonal anti-aromatase antibody (H4 clone), Ab 139492 (Abcam, Cambridge, MA, USA). The ImmPress Polymer Staining System (Vector, Burlingame, CA, USA) was used to conjugate the antibody for enzymatic detection. ImPACT DAB (Vector, Burlingame, CA, USA) was used as a chromogen for visualization. Paraffin-embedded MCF-7 and MDA-MB-231 cells were analyzed for estrogen receptor expression by the clinical Stanford Immunodiagnostic Service using the anti-estrogen receptor (ER) (SP1) rabbit monoclonal antibody in a Ventana automated slide stainer with Ventana detection kits and reagents.

### Statistics

**Fig. 4:**
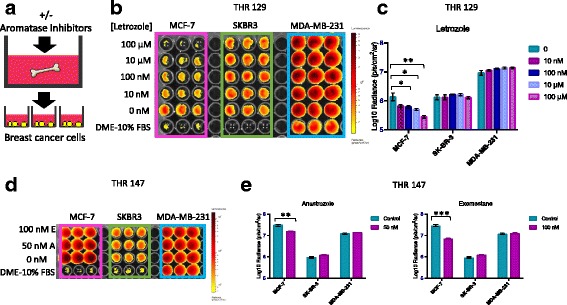
Bone tissue culture supernatants generated in the presence of aromatase inhibitors (AIs) have diminished capacity to promote estrogen receptor-positive (ER+) breast cancer cell proliferation. **a** Experimental design in which bone tissue fragments were cultured in the presence vs. absence of AIs for 48 h. **b** Bioluminescence imaging (BLI) signal displayed by breast cancer cells growing in the presence of conditioned media generated by bone fragments isolated from total hip replacement (THR) specimen 129 and cultured in DMEM-10%FBS plus 100 μM, 10 μM, 100 nM, and 10 nM, vs. 0 μM letrozole. **c** Averaged triplicate BLI signal detected for each cell line shown in (**b**). BLI signal was reduced for ER+ MCF-7, but not ER- SK-BR-3 or MDA-MB-231 cells cultured with bone tissue-conditioned media generated in the presence of 100 μM (**p = 0.003), 10 μM (*p = 0.016), 100 nM (*p = 0.035), and 10 nM (*p* = 0.056) vs. 0 μM letrozole, as determined by *t* test (*n* = 3, error bars represent standard deviation). **d** BLI signal displayed by breast cancer cells growing in the presence of conditioned media generated by bone fragments isolated from THR specimen 147 in the presence of 100 nM exemestane and 50 nM anastrozole vs. no agent. **e** Averaged triplicate BLI signal detected for cells shown in (**d**). BLI signal was specifically reduced for ER+ MCF-7 cells, but not ER- SK-BR-3 or MDA-MB-231 breast cancer cells cultured with bone tissue-conditioned media generated in the presence of 100 nM exemestane (***p = 0.00003) and 50 nM anastrozole (**p = 0.002) vs. no agent, as determined by *t* test (*n* = 3, error bars represent standard deviation)

**Fig. 5:**
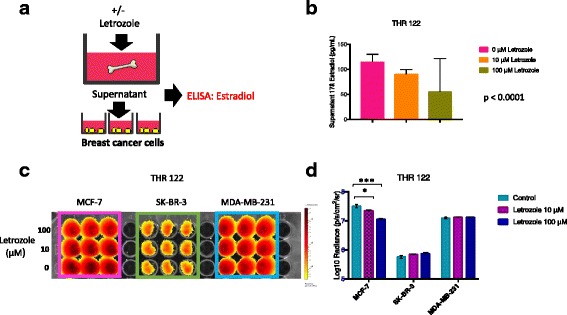
Bone tissue culture supernatants generated in the presence of aromatase inhibitors have reduced estrogen levels and diminished capacity to promote estrogen receptor-positive (ER+) breast cancer cell proliferation. **a** Experimental design in which bone tissue fragments were cultured in the presence vs. absence of letrozole for 48 h. Supernatants were used to culture breast cancer cells growing on plastic, and were also analyzed for estradiol levels by ELISA. **b** ELISA analysis of estradiol levels in conditioned media generated by bone tissue fragments isolated from total hip replacement (THR) specimen THR 122 in the presence of 100 μM and 10 μM vs. 0 μM letrozole. The lowest estradiol levels were observed in supernatants generated in 100 μM, with higher values observed in supernatants generated in 10 μM and 0 μM letrozole, corresponding to estradiol levels of 47.5, 90.8, and 114.9 pg/mL, respectively (*p* < 0.0001) as determined by analysis of variance with the Brown-Forsythe test. **c** Bioluminescence imaging (BLI) signal detected for ER+ MCF-7, and ER- SK-BR-3 and MDA-MB-231 cells cultured in replicate supernatants generated by THR 122 fragments in the presence of 100 μM, 10 μM, and 0 μM letrozole. **d** Averaged triplicate BLI signal detected for each cell line shown in **c**. BLI signal was significantly reduced in ER+ MCF-7 cells, but not ER- SK-BR-3 or MDA-MB-231 cells cultured with conditioned media generated in the presence of 100 μM (***p = 0.0008) or 10 μM (*p = 0.03) vs. 0 μM letrozole, as determined by *t* test (*n* = 3, error bars represent standard deviation)

**Fig. 6:**
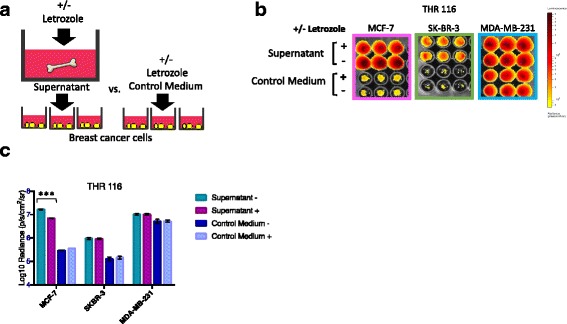
Letrozole inhibits estrogen receptor-positive (ER+) breast cancer cell proliferation indirectly by acting on bone tissues. **a** Experimental design for comparing relative breast cancer cell numbers during culture with bone tissue supernatants generated in the presence of letrozole vs. control medium containing letrozole. **b** Bioluminescence imaging (BLI) signal displayed by ER+ MCF-7, and ER- SK-BR-3 and MDA-MB-231 breast cancer cells growing in the presence of conditioned media generated by total hip replacement (THR) specimen THR 116 tissue fragments cultured in DMEM-10%FBS +/− 100 μM letrozole vs. control media (DMEM-10%FBS) +/− 100 μM letrozole. **c** Averaged triplicate BLI signal detected on plate shown in (**b**). BLI signal was specifically reduced in MCF-7, but not SK-BR-3 or MDA-MB-231 cells cultured with bone tissue culture supernatants generated in the presence of 100 μM vs. 0 μM letrozole μM (***p = 0.00003), as determined by *t* test (*n* = 3, error bars represent standard deviation). No difference was observed for any of the cell lines when 100 μM letrozole was added directly to the control medium

**Fig. 7:**
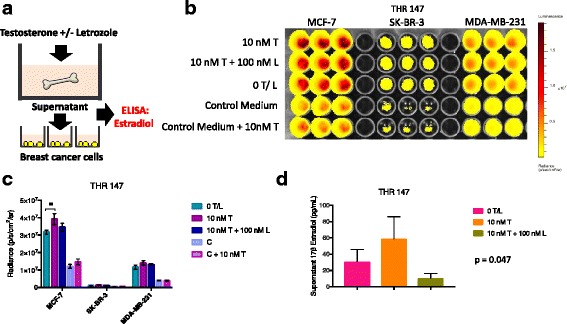
Bone tissue culture supernatants generated in the presence of testosterone have elevated estrogen levels and increased capacity to promote estrogen receptor-positive (ER+) breast cancer cell proliferation. **a** Experimental design in which bone tissue fragments are cultured in the presence of phenol red-free medium with 10% charcoal-stripped FBS containing testosterone in the presence vs. absence of letrozole. Supernatants were harvested and used to culture breast cancer cells growing on plastic and analyzed for estradiol levels via ELISA. **b** Bioluminescence imaging (BLI) signal (non-log) detected for ER+ MCF-7, and ER- SK-BR-3 and MDA-MB-231 cells cultured with supernatants generated by bone fragments isolated from total hip replacement (THR) specimen 147 in the presence of 10 nM testosterone +/− 100 nM letrozole, vs. 0 nM testosterone/letrozole. Each cell line was also cultured in control medium (phenol red-free medium with 10% charcoal-stripped FBS) in the presence vs. absence of 10 nM testosterone. **c** Averaged triplicate BLI signal detected on plate shown in **b**. BLI signal was significantly increased for ER+ MCF-7 cells, but not ER- SK-BR-3 or MDA-MB-231 breast cancer cells cultured with bone tissue-conditioned medium generated in the presence of 10 nM testosterone, as determined by *t* test (*n* = 3, error bars represent standard deviation). Although signal was reduced in the presence of 100 nM letrozole, the reduction was not significant. **d** ELISA analysis of triplicate aliquots of conditioned media collected from cultures of bone tissue fragments, showing elevated levels of estradiol in supernatants generated in the presence of 10 nM vs. 0 nM, or 10 nM testosterone + 100 nM letrozole (56.8 vs. 30.8 and 10.6 pg/mL, respectively) (*p = 0.047), as determined by analysis of variance, where *n* = 3 and error bars represent standard deviation

All statistical analyses were performed using GraphPad Prism Software, Version 7.0a. Because bioluminescence data are not normally displayed on the same scales, all BLI signal data sets were transformed to log10 values prior to statistical analysis and graphing. Data sets were graphed using log10 values in all figures except where non-log values illustrated the data more clearly. Since different cell lines express the luciferase reporter at different levels, BLI signal on a per cell basis varies across cell lines. Therefore, in experiments involving multiple cell lines, all statistical comparisons were made across averaged triplicate values for a given cell line under different conditions or treatments using multiple two-sided *t* tests. Correction for multiple *t* tests was performed using the Holm-Sidak method, with alpha = 0.05. Corrections for six multiple tests were made for data shown in Fig. 2d, and for three multiple tests per condition or treatment for data shown in Figs. 3, 4, 5, 6 and 7. When comparisons were made for a single cell line across multiple THR specimens, as in Figs. 2e and 3e, one-way analysis of variance (ANOVA) with repeated measures was performed, with alpha = 0.05, where *p* values and 95% confidence intervals for the difference between the means are reported. Error bars on all graphs represent standard deviation, with n = 3. Comparisons of estrogen levels in bone-conditioned media generated in the presence of various treatments were analyzed using one-way ANOVA with alpha = 0.05, with use of the Brown-Forsythe test for the data shown in Fig. 5b.

## Results

### Femoral head specimens harbor trabecular bone tissues for modeling the breast cancer-to-bone metastatic niche

Total hip replacement is most commonly performed due to osteoarthritis involving the articular surface cartilage of the femoral head, and less commonly due to avascular or idiopathic osteonecrosis. These pathologic conditions do not typically involve the trabecular bone tissue located in the neck of the femoral head used in our co-culture model, as outlined in Fig. 1a–e. To confirm this, H & E stained histologic sections of trabecular bone tissues isolated from patients with either diagnosis were prepared. Figure 1e shows tissues isolated from seven patients diagnosed with osteoarthritis (THRs 122, 129, 133, 135, 139, 147, and 161), and from one patient diagnosed with osteonecrosis (THR 151), illustrating typical mineralized and marrow compartment histology. Interestingly, these tissues display the well-established pattern of increased adipose tissue typically observed in the marrow compartment throughout the skeleton with increasing age [29, 30]. Tissues from male patients were collected because our initial observations, generated during another line of experimentation, revealed that ER+ breast cancer cells were preferentially sustained in tissues from male and female patients. In this study, specimens were collected from surgical patients aged 43 to 83 years (average = 62.7), an age range that brackets the years during which peak breast cancer incidence occurs. In fact, medical record review revealed that two of the specimens were isolated from female patients diagnosed with breast cancer within the year prior to (THR 116), or following (THR 122) hip replacement surgery. Thus, the tissues used for these studies represent the aging skeleton that is targeted by bone-seeking malignancies during metastasis.

**Fig. 8:**
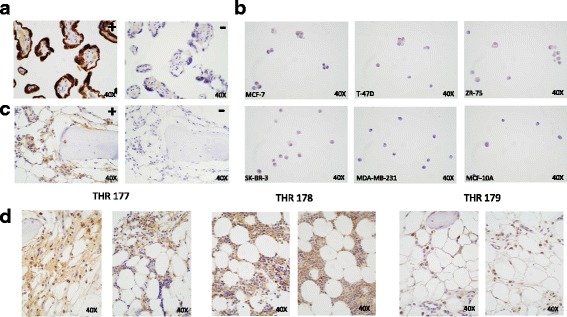
Bone tissues express aromatase. Immunostaining with anti-aromatase H4 monoclonal antibody detects aromatase expression in the bone marrow compartment of trabecular bone tissue fragments. **a** Positive control placental tissue stained with (+) vs. without (−) primary antibody. **b** Paraffin-embedded breast cancer cell lines ER+ MCF-7, T-47D, ZR-75, and ER- SK-BR-3, MDA-MD-231, and MCF-10A stained with primary antibody. **c** Trabecular bone tissues from total hip replacement (THR) specimen 177 stained with (+) vs. without (−) primary antibody. **d** Additional bone tissues from THRs 177, 178 and 179 stained with primary antibody

### Human bone tissues preferentially sustain ER+ vs. ER- breast cancer cells

To study the responses of breast cancer cell lines during short-term co-culture with human bone tissue fragments, cell suspensions prepared from six breast cancer cell lines were seeded at low density into tissue culture wells vs. directly onto trabecular bone tissue fragments isolated from a given THR specimen, as illustrated in Fig. 2a, and monitored with BLI to compare relative cell numbers over time. Under either of these conditions, the overall cell numbers of most cell lines declined from day 2 to day 4, reflecting a net loss of viable cells (Additional file 1: Figure S1). However, an experiment performed using bone fragments from a 50-year old female patient (THR 151) revealed that ER+ MCF-7, ZR-75, and T-47D breast cancer cells exhibited dramatically greater BLI signal, reflecting higher numbers of viable cells, following co-culture on bone tissue fragments vs. plastic, as shown in Fig. 2b. The ratio of BLI signal on day 4 of culture on bone tissue fragments vs. on plastic was greatest for the ER+ cell lines MCF-7 (83.5x), ZR-75 (15.4x), and T47-D (18.3x), intermediate for the ER-/human epidermal growth factor receptor 2 (HER2) + SK-BR-3 cells (9.2x), and lowest for the ER- MDA-MB-231 (0.5x) and MCF-10A (0.3x) breast cells, as shown in Fig. 2c. Statistical analysis of log-transformed data using multiple two-sided *t* tests (with correction for six tests) revealed that BLI signals were significantly greater during culture on bone vs. plastic for the three ER+ cell lines (MCF-7 (*p* < 0.001), ZR-75 (*p* < 0.01), T-47D (*p* < 0.05)), and the ER-/Her2+ cell line (SK-BR-3 (*p* < 0.01)). No difference was detected on bone vs. plastic for the ER- MDA-MB-231 cells. BLI signal was significantly reduced on bone vs. plastic for the ER-negative breast cell line MCF-10A (*p* < 0.05) (Fig. 2d). These patterns were also observed when identical experiments were performed testing MCF-7, SK-BR-3, and MDA-MB-231 cells across a series of 12 THR specimens collected from male and female hip replacement surgery patients, as shown in Fig. 2e. One-way repeated measures ANOVA demonstrated significantly greater BLI signal for culture on bone vs. plastic for MCF-7 cells (*p* = 0.0053; 95% CI (0.1881 to 0.8424)), whereas BLI signal was significantly reduced for SK-BR-3 and MDA-MB-231 cells (*p* = .0184; 95% CI (−0.3807 to −0.04318)) and (*p* = 0.0003; 95% CI (−0.9934 to −0.4184)), respectively. These results suggest that the human bone tissue microenvironment of the female and male skeleton preferentially sustains the viability of ER+ breast cancer cells compared to ER- breast cancer cells seeded directly onto human bone tissue fragments.

### Bone tissue-conditioned culture media preferentially enhance ER+ vs. ER- breast cancer cell proliferation

To determine if ER+ breast cancer cells are preferentially sustained by one or more soluble factors released into the bone microenvironment, breast cancer cells were seeded directly into plastic culture dishes and cultured in bone tissue-conditioned medium vs. control medium (DMEM-10%FBS), as shown in Fig. 3a, and BLI signal was monitored over time. Under these conditions, MCF-7 ER+ cells exhibited increased proliferation, as reflected by net increases in viable cells, in the presence of bone tissue-conditioned medium vs. control medium (Additional file 2: Figure S2). In an initial experiment in which conditioned medium was generated using tissues from a 57-year old male patient (THR 133), BLI on day 4 of culture revealed increased signal in ER+ MCF-7 and ER- SK-BR-3 cells, but not ER- MDA-MB-231 cells cultured in bone tissue supernatants vs. control medium, as shown in Fig. 3b. The ratio of BLI signal generated during culture in bone tissue-conditioned medium vs. control medium was greatest for the ER+ MCF-7 cell line (40.1x), and comparatively lower for the ER- SK-BR-3 (2.8x), and MDA-MB-231 (1.4x) cells (Fig. 3c). Statistical analysis of log-transformed data using multiple two-sided *t* tests, with correction for three comparisons, revealed that BLI signal was significantly greater during culture in bone tissue-conditioned vs. control medium for MCF-7 (*p* = 0.0000028) and SK-BR-3 (*p* = 0.0046), but not MDA-MB-231 cells (Fig. 3d). These patterns were also observed when identical experiments were performed testing MCF-7, SK-BR-3 and MDA-MB-231 cells across supernatants generated by a series of eight THR specimens collected from male and female hip replacement surgery patients (Fig. 3e). One-way repeated measures ANOVA demonstrated significantly greater signal for MCF-7 and SK-BR-3 cells cultured in bone-conditioned vs. control media (*p* = 0.0004; 95% CI (0.9147 to 2.007)) and (*p* = 0.0142; 95% CI (0.1855 to 1.183)), but not for MDA-MB-231 cells (*p* = 0.8821; 95% CI (−0.6493 to 0.5721)). These results suggest that one or more soluble factors from the human bone tissue microenvironment preferentially promotes the proliferation of ER+ vs. ER- breast cancer cells.

### Pretreatment of bone tissues with aromatase inhibitors reduces the capacity of conditioned media to promote ER+ breast cancer cell proliferation

As ER+ breast cancer cells were preferentially sustained by cultured bone tissue fragments and conditioned media, we hypothesized that the bone tissues synthesize estrogen. In a first step to test this, human bone tissue fragments were cultured in the presence vs. absence of aromatase inhibitors, and the supernatants were used to culture ER+ and ER- breast cancer cell lines, as shown schematically in Fig. 4a. In an initial experiment using tissues from a 59-year old male patient (THR 129), bone fragments were cultured in DMEM-10% FBS plus 100 μM, 10 μM, 100 nM, and 10 nM, vs. 0 μM letrozole, and BLI was performed after 4 days of culture to assess relative breast cancer cell numbers. As shown in Fig. 4b and c, BLI signals were significantly reduced for ER+ MCF-7, but not ER- SK-BR-3 or MDA-MB-231 cells cultured with bone tissue-conditioned media generated in the presence of 100 μM (*p* = 0.003), 10 μM (*p* = 0.016), 100 nM (*p* = 0.035), and 10 nM (*p* = 0.056) vs. 0 μM letrozole. To determine if this response could be observed using other aromatase inhibitors, tissue fragments from a 62-year old female patient (THR 147) were cultured in the presence vs. absence of exemestane or anastrazole to generate supernatants. As shown in Fig. 4d and e, ER+ MCF-7, but not ER- SK-BR-3 or MDA-MB-231 exhibited reduced BLI signals when cultured in supernatants generated in the presence of 100 nM exemestane (*p* = 0.00003) or 50 nM anastrazole (*p* = 0.002) vs. no agent. These results demonstrate that pretreatment of bone tissues isolated from both male and female patients, with three different types of aromatase inhibitors, reduces the capacity of culture supernatants to promote ER+ breast cancer cell proliferation in a dose-dependent manner.

### Bone tissue culture supernatants generated in the presence of aromatase inhibitors have reduced estrogen levels in association with diminished capacity to sustain ER+ breast cancer cell proliferation

To determine if pretreatment of bone tissues with aromatase inhibitors also reduced estrogen levels, bone tissue fragments isolated from a 43-year old female patient (THR 122) were cultured in the presence vs. absence of letrozole. Aliquots of these supernatants were tested for their ability to promote breast cancer cell proliferation by monitoring relative cell numbers with BLI and were also analyzed for estrogen levels by ELISA, as shown schematically in Fig. 5a. ELISA analysis (performed with the B-Bridge kit) detected 17β-estradiol concentrations of 114.9, 90.8, and 45.7 pg/mL in supernatants generated in the presence of 0, 10, and 100 μM letrozole, respectively (Fig. 5b). In keeping with these observed 17β-estradiol supernatant levels, BLI signal on day 4 of culture was significantly reduced in ER+ MCF-7 cells, but not ER- SK-BR-3 or MDA-MB-231 cells cultured with conditioned media generated in the presence of 100 μM (*p* = 0.0008) or 10 μM (*p* = 0.03) vs. 0 μM letrozole. These responses are consistent with previous data demonstrating estrogen receptor expression and proliferative response to estrogen in culture by MCF-7, but not MDA-MB-231 cells (Additional file 3: Figure S3). The level of estrogen released into the experimentally determined 5 mL volumes of tissue culture medium does not represent the actual concentrations within the bone tissue microenvironment. We used two different ELISA kits to analyze supernatants (as described in “Methods”). Using the first kit (B-bridge), which has a sensitivity of 39.6 pg/mL and lower limit of detection of 26.5 pg/mL, we detected estradiol in only one of three samples (THR 122, 114.90 pg/mL), demonstrating modulation of these levels with letrozole. We thus employed a second kit (Cayman), which has a sensitivity of 25 pg/mL and lower limit of detection of 6.6 pg/mL, and detected estradiol in four of four (or 4/4) samples, including THR 141 (35.99 pg/mL), THR 147 (30.79 pg/mL), THR 154 (25.79 pg/mL), and THR 155 (11.78 pg/mL). These levels of 17β-estradiol observed in supernatants are consistent with levels used in cell culture to stimulate MCF-7 cell proliferation [31]. Importantly, the relative 17β-estradiol levels detected in THR 122 supernatants correlated with modulation of ER+ breast cancer cell responses by aromatase inhibitors. These results demonstrate that pretreatment of bone tissues with letrozole reduces the capacity of supernatants to promote ER+ breast cancer cell proliferation in association with lowered 17β-estradiol levels.

### Letrozole inhibition of ER+ breast cancer cells is mediated by bone tissues

To determine if residual letrozole in the conditioned bone tissue supernatants inhibits breast cancer cell proliferation directly in our model, breast cancer cells were cultured in the presence of bone tissue supernatants generated in the presence of letrozole vs. control medium (DMEM-10%FBS) containing equal concentrations of letrozole, as shown schematically in Fig. 6a. In this experiment, using tissue fragments isolated from a 50-year old female patient (THR 116), BLI signals on day 4 were specifically reduced in ER+ MCF-7, but not ER- SK-BR-3 or MDA-MB-231 cells cultured with supernatants generated in the presence vs. absence of 100 μM letrozole, as shown in Fig. 5b and c. However, no differences were observed for any of the cell lines cultured in control medium containing 100 μM letrozole. These results demonstrate that the inhibitory effects of letrozole in this model system are mediated by bone tissues, as opposed to acting directly on the ER+ breast cancer cells.

### Bone tissue culture supernatants generated in the presence of testosterone have increased estrogen levels and increased capacity to sustain ER+ breast cancer cell proliferation

Estrogens are synthesized through the conversion of the precursor androgens androstenedione and testosterone. To determine if levels of estrogen were increased in the bone tissue supernatants following the addition of the estrogen precursor testosterone, bone tissue fragments from a 62-year old female patient (THR 147) were cultured in the presence of phenol red-free medium with charcoal-stripped 10% FBS containing 10 nM testosterone +/- 100 nM letrozole vs. 0 nM testosterone, as shown schematically in Fig. 7a. Supernatants were harvested, analyzed for 17β-estradiol levels via ELISA using the Cayman kit, and used to culture breast cancer cells growing on plastic. The 17β-estradiol levels in supernatants generated in the presence of 0 nM testosterone, 10 nM testosterone, and 10 nM testosterone + 100 nM letrozole were 30.8, 56.8, and 10.6 pg/mL, respectively. In cell culture experiments using replica aliquots of these supernatants, ER+ MCF-7, but not ER- SK-BR-3, and MDA-MB-231 cell BLI signal increased during culture with supernatants generated in the presence of 10 nM vs. 0 nM testosterone (*p* = 0.042). The addition of 100 nM letrozole to testosterone was associated with a reduction in MCF-7 BLI signal, but the reduction was not statistically significant. This experiment suggests that cultured human bone tissue fragments in this model system have the capacity to convert the precursor steroid testosterone into estrogen.

### Aromatase expression is detected in the bone marrow compartment of trabecular bone tissues

To determine if the trabecular bone tissue fragments used in our model system express the aromatase enzyme, a subset of tissues were immunohistochemically analyzed using the H4 clone aromatase monoclonal antibody, which detects a single protein band at ~55 kDa in western blot analysis [32]. As shown in Fig. 8a, robust expression was observed in the positive control placental tissue. Extremely low levels of expression were detected in paraffin-embedded sample preparations of the ER+ MCF-7, T-47D and ZR-75 cells and ER- SK-BR-3 cells, whereas the ER- MDA-MB-231 and MCF-10A breast cells were negative for expression, as shown in Fig. 8b. Intermediate to strong staining was detected throughout the marrow compartment of trabecular bone tissue fragments from THRs 177, 178, and 179, as illustrated in Fig. 8c and d. Although the identity of positive cells was not addressed in the current study, staining was observed within hematopoietic and stromal regions, including the bone marrow adipose tissue. No staining was detected within the mineralized compartment. These results are consistent with previously published studies [33], and demonstrate that the aromatase enzyme is expressed by the bone marrow compartment of the human trabecular bone tissues used in our model system. The extremely low and negative level of aromatase expression observed in all the breast cancer cell lines are consistent with our data showing that addition of letrozole directly to cell line cultures did not inhibit proliferation.

## Discussion

This investigation began when we observed a differential pattern of breast cancer cell colonization in our human bone tissue co-culture model system. While MCF-7 cell viability was enhanced during co-culture on bone tissue fragments compared to plastic culture dishes, this relative response was less pronounced in SK-BR-3 cells, and diminished in MDA-MB-231 cells. Extension of these comparisons to the additional ZR-75, T-47D, and MCF-10A breast cell lines suggested that, as a group, ER+ breast cancer cell lines are preferentially sustained on bone fragments relative to plastic, compared to ER- breast cancer cell lines. Additional experiments demonstrated a differential proliferative response of ER+ breast cells to bone tissue-conditioned vs. control media, suggesting that this preference is at least partially mediated by soluble factors. Both patterns were consistently observed across an extended series of bone tissue specimens isolated from a series of male and female patients undergoing hip replacement surgery. The enhanced viability and proliferative responses of ER+ vs. ER- breast cancer cells during short-term co-culture with human bone tissue fragments and conditioned media are consistent with long-established clinical patterns demonstrating a propensity for ER+ breast cancer to preferentially target the skeleton during metastatic spread [20–23]. The proclivity of ER+ breast cancer for the human skeleton suggests that factors available in the bone microenvironment provide a selective advantage for this subtype.

Although ER+ breast cancers are driven by estrogen, most breast cancer is diagnosed during the postmenopausal years when estrogen production by the ovaries declines. The major source of estrogen during these years is through the peripheral conversion of androgens into estrogen by the aromatase enzyme, which is expressed in a variety of tissues throughout the body, including brain, skin, and adipose tissue [5]. In postmenopausal women, the largest source of estrogen is adipose tissue, including subcutaneous and abdominal depots [12]. This pattern, coupled with the widely-observed association between obesity and breast cancer incidence, particularly for ER+ breast cancers, has led to the hypothesis that estrogen produced by subcutaneous and abdominal fat drives the development and progression of breast cancer [13–15]. However, many studies indicate that peripherally produced estrogen acts locally, in paracrine fashion [15]. Indeed, local estrogen production is implicated in the development of primary breast tumors, where estrogen concentrations have been found to exceed circulating levels by as much as ten times [7, 8]. Aromatase expression is higher in adipose tissue adjacent vs. distal to primary tumors, suggesting local provision of estrogens to fuel primary tumor progression [9–11, 34].

Aromatase expression and estrogen biosynthesis also occur throughout the human skeleton [5]. Frisch et al. reported the production of estrogens from androgens in fatty marrow extracts isolated from femoral heads and iliac crests obtained from female subjects undergoing orthopaedic surgery [35]. Schweikert and Romalo demonstrated aromatase enzymatic activity and estrogen production in extracts prepared from ground trabecular bone tissues isolated from specimens obtained following hip replacement surgeries in male and female patients, and reported that the addition of aromatase inhibitors to these extracts inhibited estrogen production [36]. Sasano et al. evaluated aromatase immunoreactivity and RNA expression via in situ hybridization and RT-PCR in bone tissue specimens including ribs, vertebrae and femurs from a series of 28 male and female patients undergoing orthopaedic surgery [33]. In this study, aromatase RNA expression was detected by RT-PCR in 26/28 of the specimens, and aromatase protein and RNA expression were localized to osteoblasts, osteocytes, chondrocytes and adipocytes. Aromatase activity and in situ estrogen production has also been detected in cultured osteoblasts isolated from human bone tissues by Nawata et al. [37], and other studies have revealed the presence of estrogen receptors in osteoblasts [38, 39]. Together, these studies provide solid evidence demonstrating a local estrogen axis within the skeleton. A central physiological role for estrogen in bone tissue maintenance is implicated by skeletal phenotypes observed in association with aromatase deficiency in aromatase knockout mice (arKO) [40] and aromatase gene mutations in humans, including loss of bone mass and osteoporosis in male and female subjects (reviewed, [41]). Collectively, these findings by others suggest that estrogen biosynthesis occurs locally in bone, and that estrogen is required to sustain a mineralized skeleton in women and men.

Our study suggests that local estrogen production within the bone microenvironment specifically regulates ER+ breast cancer cells. The trabecular bone tissues used in our model represent the microenvironment of the aging skeleton encountered by disseminated tumor cells in bone-seeking malignancies. In vivo metastasis experiments in a mouse model have shown that the majority of disseminated breast cancer cells initially reaching the bone marrow compartment are cleared within the first 24 h [42], corroborating metastatic inefficiency observed in other metastatic sites [43–45]. In the mouse model, small numbers of remaining cells commenced proliferation after several days to establish micrometastases. In our ex vivo model, the numbers of viable breast cancer cells steadily dropped during short-term co-culture on bone tissue fragments. However, the ratio of viable cells after 4 days of culture on bone tissues vs. plastic was dramatically higher for ER+ cells compared to ER- cells. This suggests that bone tissues sustain a higher level of survival for ER+ vs. ER- breast cancer cells during initial colonization events.

Experiments performed in bone-conditioned media suggest that in addition to enhancing viability, locally produced estrogen also sustains ER+ breast cancer cell proliferation, and that aromatase inhibitors directly modulate this axis. In vitro, estrogen-induced responses are highly dependent on culture conditions. Most commonly, estrogen-induced proliferation has been reported in cultures containing 10^-12^ − 10^-7^ M estradiol, but responses are highly variable due to culture components, including serum (which contains estradiol, growth factors, and cytokines), and the pH indicator phenol red (which has estrogenic activity) [46–52]. As these conditions can potentially mask estrogen-induced responses, estrogen studies are commonly performed using charcoal-stripped serum and phenol red-free medium. Although lower concentrations of serum (1–5%) are typically used when assessing hormonal effects on cancer cells, our initial observations that ER+ breast cancer cells were preferentially sustained by bone tissues and conditioned medium emerged during co-culture experiments conducted in DMEM-10% FBS with phenol red. Estradiol levels in the FBS used in our experiments were reported by the vendor to contain 32 pg/mL, and were diluted 10-fold in medium to a concentration of 3.2 pg/mL, or ~ 10^-12^ M. Thus, in our co-culture conditions, we observed estrogen-induced responses in the presence of medium containing phenol red, containing low levels of estrogen. In our experiments, the enhanced responses to bone tissues and bone tissue-conditioned media were specific to ER+ vs. ER- cell lines, and were highly consistent across bone tissue specimens isolated from a series of male and female patients. In subsequent experiments, these responses were reproducibly inhibited by pretreatment of the cultured bone tissue fragments with three aromatase inhibitors - letrozole, anastrozole, and exemestane. This inhibition was highly specific to ER+ cell lines, was observed in a dose-response manner, and correlated with estradiol levels in conditioned media. Parallel experiments using control (non-conditioned) medium containing equivalent concentrations of AIs demonstrated that, in this model system, the effects of AI inhibition were mediated indirectly through the bone tissues as opposed to directly acting on the cell lines. In addition, elevated estradiol levels were detected in bone tissue-conditioned media generated in the presence of phenol red free-medium/charcoal-stripped serum containing the androgen precursor testosterone. And finally, aromatase expression was observed throughout the marrow compartment of trabecular tissue fragments. Taken together, our experiments extend the earlier body of work describing the local production of estrogen in the human skeleton, and demonstrate that aromatase inhibitors modulate this production to specifically inhibit ER+ breast cancer cell proliferation.

Aromatase inhibitors constitute the preferred therapeutic agent for the treatment of postmenopausal ER+ breast cancer patients, in the adjuvant setting to prevent recurrence of early-stage breast cancer, and as first-line and later agents to treat metastatic breast cancer [53]. Although these agents provide an invaluable armamentarium for fighting breast cancer progression, their efficacy is reduced by the challenges of side effects (most notably bone loss and skeletal fractures) [54] and endocrine resistance. In general, up to a third of all ER+ breast cancer patients exhibit intrinsic or acquired resistance to endocrine therapies [53]. As a first line treatment for ER+ metastatic breast cancer, aromatase inhibitors have a response rate of 30–50%, and eventually all endocrine therapies fail in the metastatic setting due to treatment resistance [16–18]. Multiple mechanisms are hypothesized as drivers of resistance, including: (i) overexpression of ER, (ii) mutation of ER (ESR1 mutations), (iii) ligand-independent activation of ER and/or co-activators, and (iv) activation of alternate growth and survival “escape” pathways, including the PI3K/Akt signaling pathway [16–18]. Recent preclinical and clinical studies have shown the potential for combining aromatase inhibitors with agents that block escape pathways mediating resistance, including taselisib (a PI3K inhibitor), everolumus (an mTOR inhibitor), and palbociclib (a CDK 4/6 inhibitor) [18, 55, 56]. Indeed, the addition of palbociclib to letrozole in the PALOMA trial resulted in the largest increase in progression-free survival (PSF) ever reported for a first-line agent, (14.5 vs. 24.8 months, respectively) [57]. Although combination therapies show great promise, so far they have not led to increased overall survival [58]. Thus, further characterization of the estrogen signaling axis and the mechanisms that underlie endocrine resistance during metastatic progression remain central challenges [58].

Regulation of the estrogen signaling axis is highly context-specific. In vivo, estrogens (and SERMS) are pleiotropic and induce tissue-specific responses, which are presumably mediated by the constellation of microenvironmental factors that stimulate signaling pathways converging on the estrogen receptor [19]. Our previous analysis of the human bone tissue secretome revealed a rich network of growth factors and cytokines implicated in tumorigenesis, including IL-1β, epidermal growth factor (EGF) and fibroblast growth factor (FGF) [26]. Interestingly, in an in vitro model, EGF and FGF have been shown to drive breast cancer cell resistance to letrozole via activation of the PI3K signaling pathway [55]. Other studies have shown that the cytokine IL-1β activates ER transcriptional activity and modulates response to 4-OH-tamoxifen, rendering it an agonist instead of an antagonist (reviewed in [59]). Other studies also suggest that stromal and immune cells within the tumor microenvironment may play a role in promoting endocrine resistance (reviewed in [59]). And finally, Bernoulli et al. have recently described a mouse model in which estrogen supplementation is required to support the growth of orthotopic ER+ MCF-7 primary tumors, but not ER+ MCF-7 lesions in the skeleton [60]. Collectively, these findings underscore the potential role of the local microenvironment in regulating estrogen signaling events and the development of endocrine resistance mechanisms.

## Conclusion

Our study demonstrates that during co-culture, human bone tissues preferentially sustain ER+ breast cancer cells, and that aromatase inhibitors effectively target the estrogen axis in bone tissues to specifically modulate the behavior of ER+ breast cancer cells. These results suggest that local estrogen signaling networks may drive the progression of ER+ breast cancer in the skeleton, influence the development of resistance to endocrine therapies, and underlie the development of skeletal side effects. Understanding the source and regulation of locally produced estrogens and their paracrine interactions in regulating breast cancer progression within the context of the end-target microenvironment will improve the efficacy of endocrine therapies.

## Additional files

Additional file 1: Figure S1. Relative breast cancer cell numbers during culture on bone tissue fragments vs. plastic. **A** BLI signal generated by six breast cancer cell lines (ER+ MCF-7, ZR-75 and T-47D, ER-/Her2+ SK-BR-3, and ER- MDA-MB-231 and MCF-10A) at 24, 48, 72, and 96 h of culture in plastic wells vs. bone tissue fragments isolated from THR 151. While direct culture of breast cancer cells on bone tissues resulted in an overall reduction in cell numbers from day 2 to 4, the ratio of BLI signal on bone vs. plastic, reflecting viable cell numbers, is greatest for ER+ breast cancer cells. **B** To confirm that this pattern did not result from seeding higher numbers of MCF-7 cells, the experiment was repeated to include BLI signal measurement on day 0 for 3 cell lines (MCF-7, SK-BR-3, and MDA-MB-231), using bone fragments from THR 221. This experiment demonstrates that higher numbers of MCF-7 cells on day 4 did not result due to seeding higher numbers of cells. This pattern suggests a survival advantage of ER+ vs ER- breast cancer cells in the bone microenvironment. For each data point, *n* = 3, with error bars representing standard deviation. (PDF 112 kb)

Additional file 2: Figure S2. Relative breast cancer cell numbers during culture in bone tissue-conditioned vs. control media. BLI generated by three breast cancer cell lines (ER+ MCF-7, ER-/Her2+ SK-BR-3, and ER- MDA-MB-231) at 24, 48, 72, and 96 h of culture in the presence of control medium (DMEM-10%FBS) vs. bone tissue-conditioned media from THRs 133, 129, 147, 122, and 116. Culture in bone tissue-conditioned media led to reduced SK-BR-3, and increased MCF-7 and MDA-MB-231 cell numbers. However, the ratio of cell numbers in the presence of conditioned vs. control media was greatest for MCF-7 cells. These patterns suggest that bone tissue-conditioned media preferentially promote ER+ vs. ER- breast cancer cell proliferation. For each data point, n = 3, with error bars representing standard deviation. (PDF 660 kb)

Additional file 3: Figure S3. Estrogen receptor expression and response to estrogen in culture. **A** Immunohistochemical staining with anti-estrogen receptor antibody detected estrogen receptor expression in MCF-7, but not MDA-MB-231 cells. **B** When cultured in phenol red-free medium with 5% charcoal-stripped serum, the addition of 100 nM estradiol elicited a proliferative response by the ER+ MCF-7, but not ER- MDA-MB-231 cells. Significantly greater BLI signal was detected after 5 days of culture in the treated vs. control cultures (*p* = 0.042) as determined by *t* test (n = 3, error bars represent standard deviation). These results confirm the estrogen-responsiveness of ER+ MCF-7 cells relative to the ER- MDA-MB-231 cells used in our model. (PDF 6849 kb)

## Abbreviations

Ab: Antibody
AI: Aromatase inhibitor
ANOVA: Analysis of variance
arKO: Aromatase knockout
ATCC: American Type Culture Collection
BLI: Bioluminescence imaging
CDK: Cyclin-dependent kinase
CO_2_: Carbon dioxide
DAB: 3,3′-Diaminobenzidine tetrahydrochloride
DMEM: Dulbecco’s modified Eagle’s medium
DMSO: Dimethylsulfoxide
EGF: Epidermal growth factor
EGFP: Enhanced green fluorescence protein
ELISA: Enzyme-linked immunosorbent assay
ER-: Estrogen receptor-negative
ER+: Estrogen receptor-positive
ESR1: Estrogen receptor 1 gene
FBS: Fetal bovine serum
FGF: Fibroblast growth factor
FLuc: Firefly luciferase
H & E: Hematoxylin and eosin
HER2: human epidermal growth factor receptor 2
IL-1β: Interleukin 1 beta
kDa: Kilodalton
mTOR: Mechanistic target of rapamycin
PFS: Progression-free survival
PI3K: phosphatidylinositol-4,5-biphosphonate 3-kinase
ROI: Region of interest
RPMI: Roswell Park Memorial Institute
RT-PCR: Reverse transcription Polymerase chain reaction
SERD: Selective estrogen receptor downregulator
SERM: Selective estrogen receptor modulator
STR: Short tandem repeat
THR: Total hip replacement
