# Supplementary figures and images for: Local estrogen axis in the human bone microenvironment regulates estrogen receptor-positive breast cancer cells
Source: Breast Cancer Res. 2017 Nov 15;19:121. doi: 10.1186/s13058-017-0910-x (PMC5688761; doi:10.1186/s13058-017-0910-x)

A

THR 151

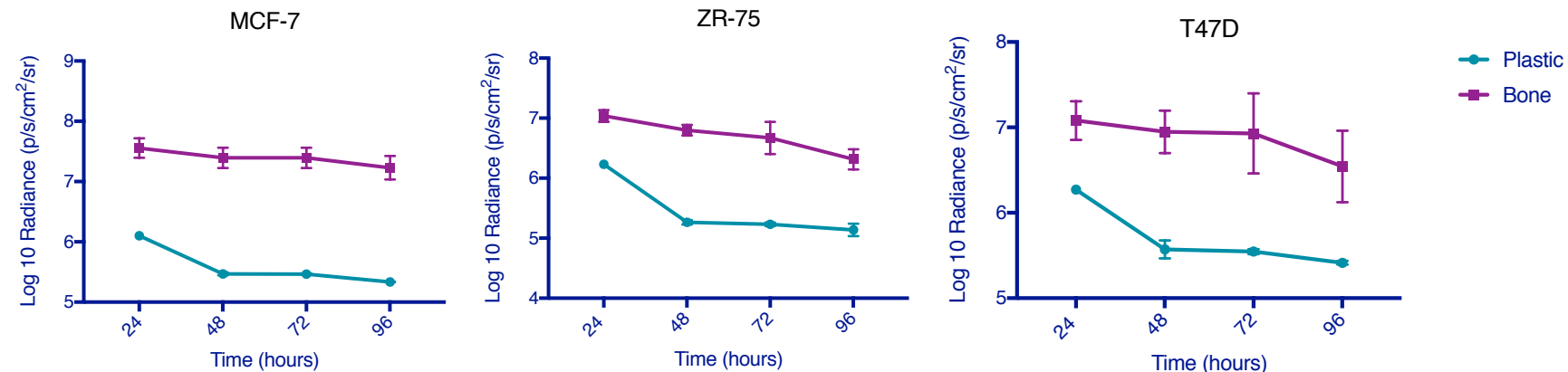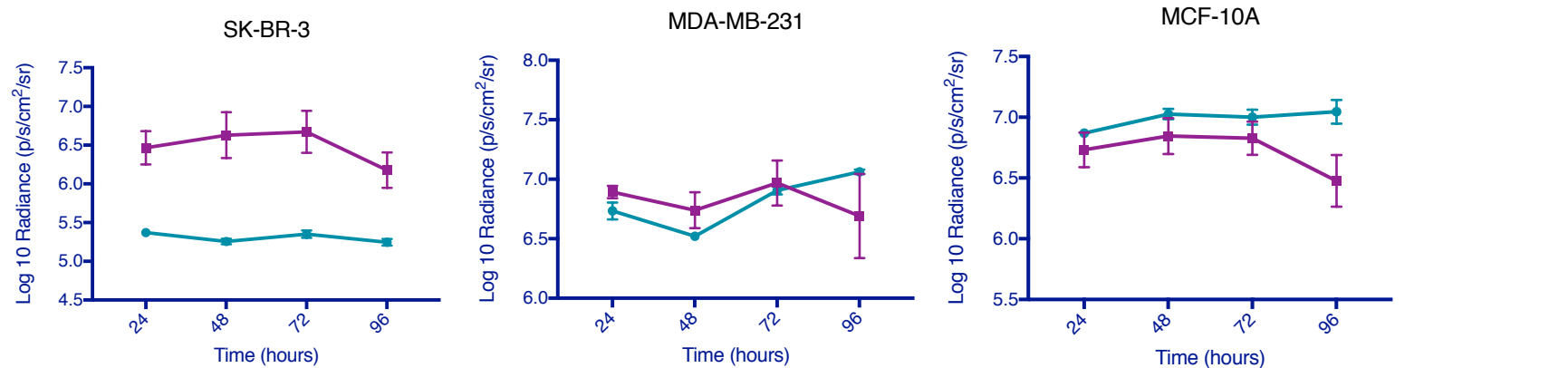

B

THR 221

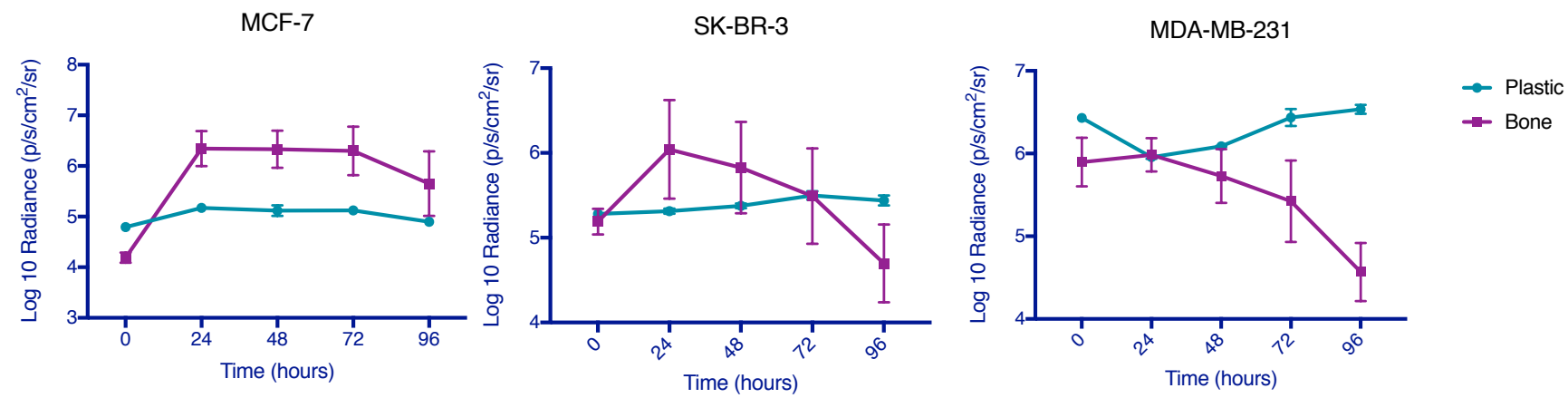

Supplement: Supplementary file 1 — Relative breast cancer cell numbers during culture on bone tissue fragments vs. plastic. A BLI signal generated by six breast cancer cell lines (ER+ MCF-7, ZR-75 and T-47D, ER-/Her2+ SK-BR-3, and ER- MDA-MB-231 and MCF-10A) at 24, 48, 72, and 96 h of culture in plastic wells vs. bone tissue fragments isolated from THR 151. While direct culture of breast cancer cells on bone tissues resulted in an overall reduction in cell numbers from day 2 to 4, the ratio of BLI signal on bone vs. plastic, reflecting viable cell numbers, is greatest for ER+ breast cancer cells. B To confirm that this pattern did not result from seeding higher numbers of MCF-7 cells, the experiment was repeated to include BLI signal measurement on day 0 for 3 cell lines (MCF-7, SK-BR-3, and MDA-MB-231), using bone fragments from THR 221. This experiment demonstrates that higher numbers of MCF-7 cells on day 4 did not result due to seeding higher numbers of cells. This pattern suggests a survival advantage of ER+ vs ER- breast cancer cells in the bone microenvironment. For each data point, n = 3, with error bars representing standard deviation. (PDF 112 kb) [file 13058_2017_910_MOESM1_ESM.pdf]

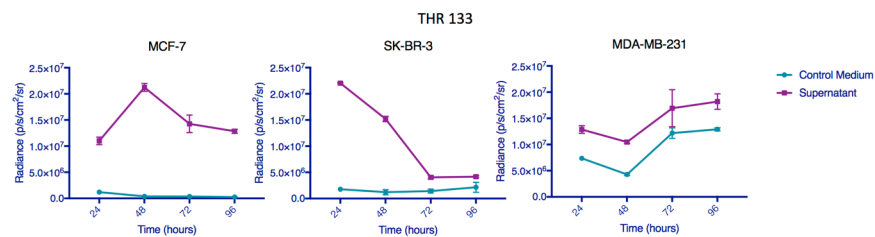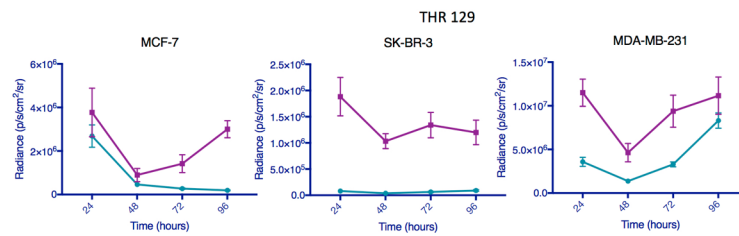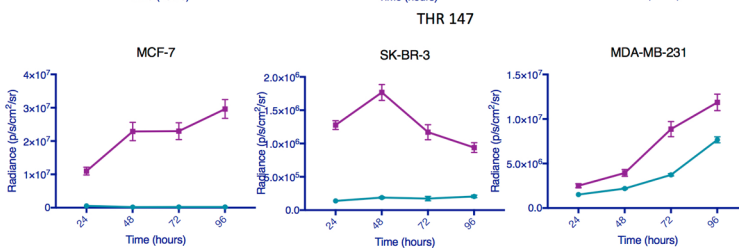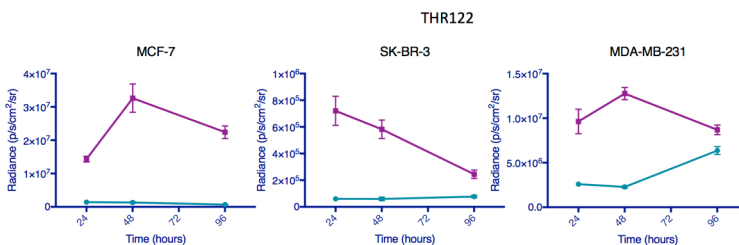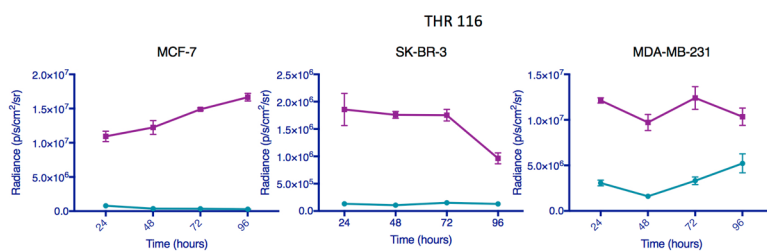

Supplement: Supplementary file 2 — Relative breast cancer cell numbers during culture in bone tissue-conditioned vs. control media. BLI generated by three breast cancer cell lines (ER+ MCF-7, ER-/Her2+ SK-BR-3, and ER- MDA-MB-231) at 24, 48, 72, and 96 h of culture in the presence of control medium (DMEM-10%FBS) vs. bone tissue-conditioned media from THRs 133, 129, 147, 122, and 116. Culture in bone tissue-conditioned media led to reduced SK-BR-3, and increased MCF-7 and MDA-MB-231 cell numbers. However, the ratio of cell numbers in the presence of conditioned vs. control media was greatest for MCF-7 cells. These patterns suggest that bone tissue-conditioned media preferentially promote ER+ vs. ER- breast cancer cell proliferation. For each data point, n = 3, with error bars representing standard deviation. (PDF 660 kb) [file 13058_2017_910_MOESM2_ESM.pdf]

A

MCF-7

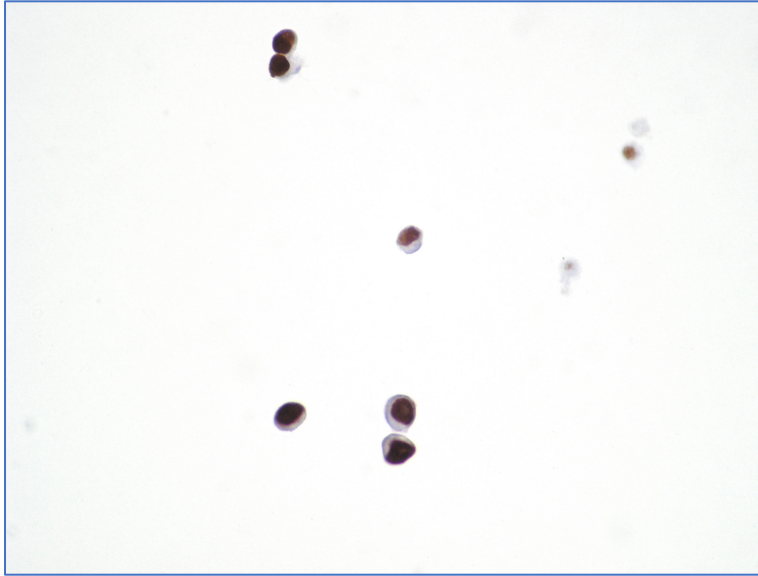

MDA-MB-231

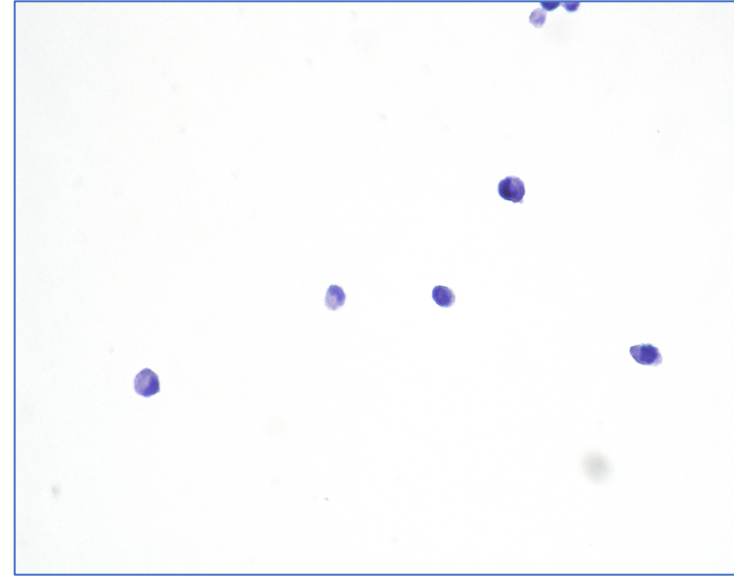

B

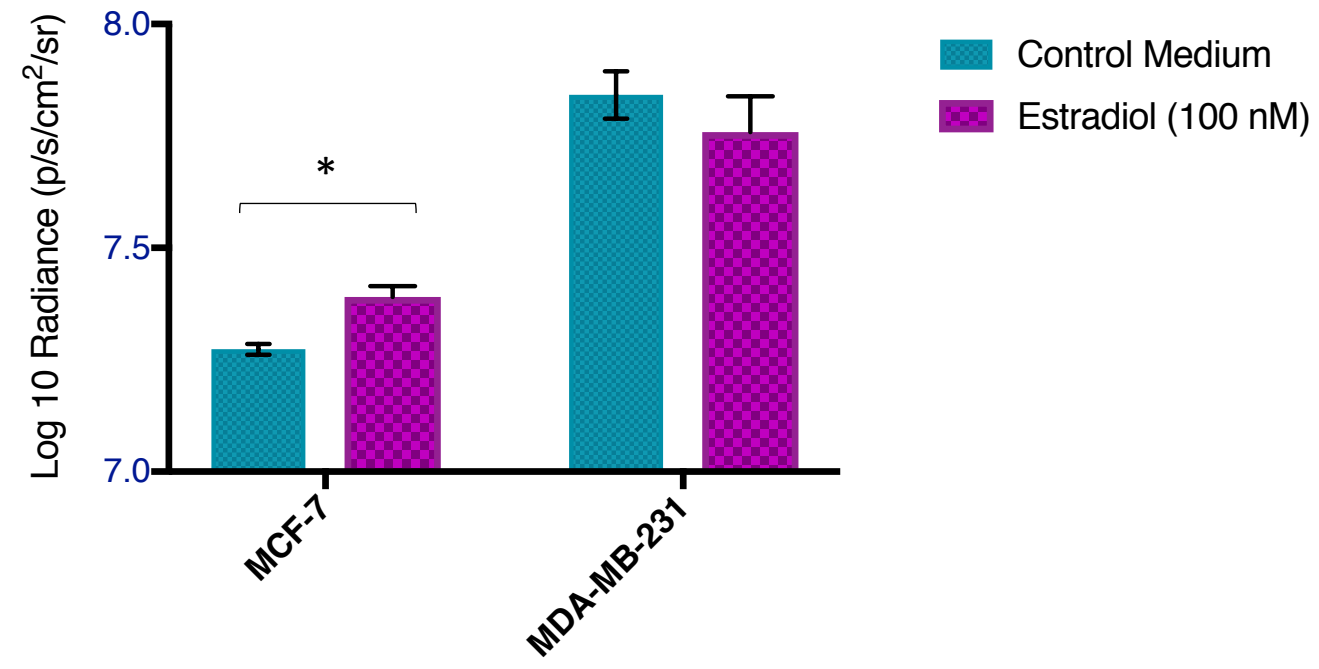

Supplement: Supplementary file 3 — Estrogen receptor expression and response to estrogen in culture. A Immunohistochemical staining with anti-estrogen receptor antibody detected estrogen receptor expression in MCF-7, but not MDA-MB-231 cells. B When cultured in phenol red-free medium with 5% charcoal-stripped serum, the addition of 100 nM estradiol elicited a proliferative response by the ER+ MCF-7, but not ER- MDA-MB-231 cells. Significantly greater BLI signal was detected after 5 days of culture in the treated vs. control cultures (p = 0.042) as determined by t test (n = 3, error bars represent standard deviation). These results confirm the estrogen-responsiveness of ER+ MCF-7 cells relative to the ER- MDA-MB-231 cells used in our model. (PDF 6849 kb) [file 13058_2017_910_MOESM3_ESM.pdf]
